# Effect of Dental Throat Pack Used Under General Anesthesia in Children with Special Healthcare Needs on Postoperative Nausea, Vomiting, and Sore Throat: A Randomized Clinical Trial

**DOI:** 10.3390/jcm14020567

**Published:** 2025-01-17

**Authors:** Sibel Tetiker, Hacer Nida Akdogan, Nilgun Alpay, Muharrem Cem Dogan

**Affiliations:** 1 Department of Anesthesiology and Reanimation, Faculty of Dentistry, Cukurova University, 01250 Adana, Turkey; nilgunalpay@yahoo.com; 2 Department of Pediatric Dentistry, Faculty of Dentistry, Cukurova University, 01250 Adana, Turkey; nidaauguz@gmail.com (H.N.A.); mcemdogan@gmail.com (M.C.D.)

**Keywords:** dental treatment, PONV, children with special healthcare needs, throat pack, sore throat

## Abstract

**Background/Objective**: Throat packs are widely used during orofacial surgery to reduce the frequency of postoperative nausea and vomiting (PONV). However, evidence supporting their use is mixed, with associated risks such as postoperative sore throat and the possibility of being forgotten in situ. **Methods**: The aim of this study is to evaluate the effectiveness of throat packs in preventing PONV and postoperative sore throat during dental treatments under general anesthesia in children with special healthcare needs (SHCNs). Eighty children with SHCNs were randomized into two groups: throat packing (Group TP, n = 41) and no throat packing (Group n-TP, n = 39). A throat pack was used in Group TP, and PONV and sore throat were evaluated at 1, 2, and 4 h postoperatively. Statistical Package for Social Sciences (SPSS) version 23.0 was used for data analysis. The registration number at ClinicalTrials.gov is NCT06169306, registered on 28 December 2023. **Results**: Patients in Group n-TP showed significantly higher PONV values at 1 and 2 h postoperatively (*p* < 0.001, *p* = 0.019, respectively). Visual Analog Scale (VAS) values were also significantly higher in Group TP at 1 and 2 h postoperatively (*p* < 0.001, *p* = 0.002, respectively). **Conclusions**: Using throat packs in dental treatments under general anesthesia for children with SHCNs reduced the incidence of PONV but increased the incidence of postoperative sore throat.

## 1. Introduction

Throat packs are widely used to prevent the swallowing and aspiration of blood and other debris, especially during orofacial surgeries and dental procedures performed under general anesthesia [1]. Postoperative nausea and vomiting (PONV) is a common complaint following surgeries performed under general anesthesia [2] and the aspiration of blood and other secretions into the stomach is a major cause of PONV. PONV reduces postoperative patient satisfaction and causes undesirable consequences, such as dehydration and electrolyte imbalance. Its incidence is more prevalent in pediatric patients than in adults, occurring in between 33% and 82% of patients [3]. There is not much evidence in the literature that throat packs prevent PONV by reducing blood aspiration, and there is no consensus among surgeons, anesthetists, or dentists regarding the application-related complications [4]. The most critical side effects associated with using throat packs are sore throat, dysphagia, and the life-threatening risk of being forgotten in situ before extubation. Death has also been reported as a major complication. Throat packs increase postoperative sore throats and cause trauma and edema in the oropharyngeal tissues [5].

According to the American Academy of Pediatric Dentistry, special healthcare needs (SHCNs) includes any physical, developmental, mental, sensory, behavioral, cognitive, or emotional disorders or limiting conditions requiring medical management, healthcare intervention, or specialized services or programs [6]. The incidence of caries and other dental problems is higher in children with SHCNs, and this is due to inadequate oral hygiene, malocclusion, high-carbohydrate diet, and lack of awareness about dental health. Safe and successful dental treatments in children with SHCNs are performed under general anesthesia; therefore, recognizing the possible side effects and complications and early intervention are essential [3]. Furthermore, it is more difficult for these patients to express and define the side effects they experience; therefore, the procedures, technical applications, and anesthesia methods performed on them must have the least potential for side effects. For surgical procedures performed under general anesthesia, PONV and sore throat have several causes besides using throat packs, such as endotracheal intubation, excessive high cuff pressure, anesthetic agents used, and gastric insufflation [7]. Therefore, ensuring the patient’s safe discharge and minimizing disturbing symptoms is essential following outpatient surgical procedures. Standardized procedural studies and reliable evidence are required to determine how much throat packing reduces or increases these adverse effects. To our knowledge, only one study has evaluated the effectiveness of postoperative throat packs in pediatric patients [8]. The necessity of routine throat packing application in children with SHCNs, along with increased possible complications due to general anesthesia, is not stated in the literature. Therefore, we investigated the effects of using throat packs during dental procedures performed under general anesthesia on PONV and sore throat in children with SHCNs. We hypothesized that using throat packs during dental treatments performed under general anesthesia in children with SHCNs does not affect PONV or postoperative sore throat.

## 2. Methods

### 2.1. Ethical Approval

This study was approved by the Cukurova University Faculty of Medicine Ethics Committee of Noninvasive Clinical Research following the Declaration of Helsinki (number 2022/118-49). The study was conducted according to the CONSORT guidelines and registered at clinicaltrials.gov (NCT06169306; 28 December 2023).

### 2.2. Sample Selection and Randomization

This randomized controlled clinical study was conducted on children with SHCNs aged 5–16 years who were uncooperative during dental treatments and were admitted to the Pediatric Dentistry Clinic of Cukurova University Faculty of Dentistry between December 2023 and April 2024.

Power analysis was performed using G Power (3.1.9.2) software. Seventy-four participants were recruited for a sample size of 37 per group, with 95% power, a 5% significance level, and effect size of 0.8. The noncentrality parameter λ value for the effect size was determined to be 3.65 and the critical t value was determined to be 1.98. The inclusion criteria were (1) volunteering to participate with parental consent, (2) age 5–16 years, (3) being in grade 1 or 2 according to the American Society of Anesthesiologists (ASA) classification [9], (4) having at least one extraction and one restorative treatment (amalgam or composite restoration), (5) having any physical, developmental, sensory, or limiting condition requiring medical management, excluding mental disorders, while being able to express themselves (e.g., epilepsy, cerebral palsy, cystic fibrosis, congenital heart disease, developmental delay, metabolic diseases).

The following exclusion criteria were applied: (1) having a tracheostomy; (2) being evaluated as difficult to intubate; (3) having esophagus-, stomach-, or intestine-related comorbidities; (4) having undergone a percutaneous endoscopic gastrostomy; (5) a history of PONV; (6) morbid obesity; (7) high airway pressure; (8) a history of allergy to the drugs to be used; (9) requiring an anesthetic agent other than the planned anesthesia method; and (10) having an intellectual disability.

**Figure 1:**
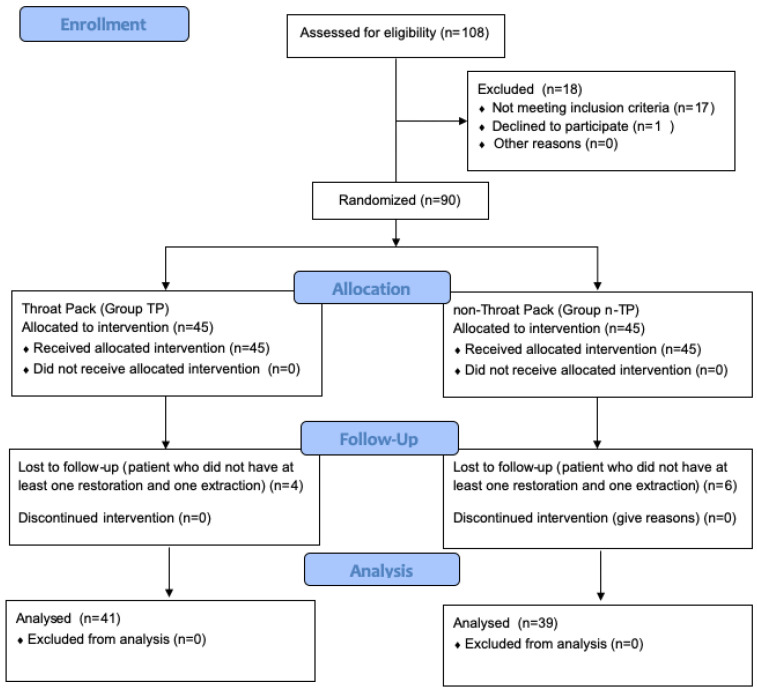
Participant flow diagram.

Notably, 108 children with SHCNs were evaluated preoperatively; however, 90 were included in the study according to the inclusion and exclusion criteria. An internet-based randomization program (researchrandomizer.org) was used in the selection of participants to avoid bias and to show that there was no difference between the groups in terms of gender and age. The 90 patients were randomly assigned into groups using a table of random numbers. Of these, 10 patients who completed dental treatments under general anesthesia but did not have at least one restoration and one extraction as specified in the inclusion criteria were excluded from the study. As a result, 41 patients in the group with throat packing (Group TP) and 39 patients in the group without throat packing (Group n-TP) were analyzed (Figure 1).

### 2.3. Procedures

All patients were preoperatively administered oral premedication with midazolam in the preoperative evaluation unit (0.5 mg/kg in children < 20 kg and 0.3 mg/kg in children > 20 kg). After premedication, the patients were transferred to the operating room with their parents. Vascular access was established with a 22–24 G branule, and an intravenous infusion of 5% dextrose and 0.45% sodium chloride (4 mL/kg/h) was administered. Standard monitoring (electrocardiography (ECG), noninvasive blood pressure (NIBP), and oxygen saturation (SpO_2_)) was performed in all patients.

General anesthesia was induced with 2 mg/kg propofol, followed by the administration of 0.5 mg/kg rocuronium as a muscle relaxant. Ventilation was provided with volume-controlled ventilation (VCV) on a mechanical ventilator, using an age-appropriate minute respiratory rate, a tidal volume of 8 mL/kg, and a maximum peak pressure of 15 cmH_2_O. Endotracheal intubation was performed by the same anesthesiologist using a Macintosh laryngoscope and an endotracheal tube (Bıçakçılar, İstanbul, Turkey) sized according to the patient’s anthropometric characteristics and measurements. The cuff inflation pressure was defined as 20 cmH_2_O using a cuff pressure indicator. This was measured using a manometer (Endotest, Teleflex Medical, Rush, Athlone, Ireland) over 30 min periods to ensure it was within the determined range. Anesthesia was maintained with a mixture of 1–2% sevoflurane and nitrous oxide/oxygen (50:50). In Group TP, the same pedodontist placed a wet throat pack consisting of one sterile, radiography-detectable gauze soaked in saline in the oropharynx using 35 × 35 mm forceps. The gauze was fixed to the mouth using a sterile 1.0 silk suture to minimize the possibility of being forgotten and was recorded on the anesthesia safety form. Throat packs were not used in the patients in Group n-TP.

The same pedodontist performed all dental treatments, and all patients underwent periodontal scaling before other dental procedures were initiated. Tooth extractions were performed after the restorative procedures were completed. A local anesthetic solution (Maxicaine Fort 40 mg/mL 0.01 mg/mL) was used in all dental treatments. The same dental nurse assisted the operator during the procedures. The extraction sites were sutured, and bleeding was controlled using a hemostatic agent.

Methylprednisolone (1 mg/kg) was administered perioperatively, and paracetamol (10 mg/kg) was administered for postoperative analgesia. After the dental treatment, the throat pack was removed from patients in Group TP. Anesthesia was noted on the security form and was terminated in all cases. Recurrence was achieved with sugammadex (2 mg/kg). Before extubation, blood in the gastric contents was observed through orogastric decompression and recorded. Once consciousness and spontaneous breathing were regained, patients were extubated and transferred to the postoperative unit. Once fully awake, the patients were transferred to the outpatient clinic and monitored for 4 h [10]. A blinded anesthesiologist who did not perform the operation scored PONV and sore throat in all patients in both groups at 1, 2, and 4 h postoperatively. Patients were discharged with values ≥ 9 according to the Modified Aldrete Scoring System [11]. Sore throat scores after endotracheal extubation were recorded at 1, 2, and 4 h using the Visual Analog Scale (VAS) score between 0 and 10. On the VAS, 0 indicates no pain, whereas 10 indicates the most severe pain. PONV scores were recorded at 1, 2, and 4 h using the pictorial nausea scale “Baxter Retching Faces (BARF)” [12], scored between 0 and 10. A score of 0 on the BARF scale indicates the absence of PONV, whereas a score of 10 indicates the most severe PONV. After the 4th postoperative hour, patients with a VAS score > 5 were scheduled to receive paracetamol (10 mg/kg). Patients with a BARF score > 4 were scheduled to receive 0.15 mg/kg ondansetron.

### 2.4. Statistical Analyses

Data analysis was conducted using the Statistical Package for the Social Sciences (SPSS) version 23.0. Categorical variables were summarized using frequencies and percentages, whereas continuous variables were presented as means and standard deviations (medians and ranges were provided where necessary). The Shapiro–Wilk test was used to assess the normality of the distribution of the variables included in the study. Categorical data were analyzed using the chi-square test. The Mann–Whitney U test was used for variables that did not follow a normal distribution. Statistical significance was set at a *p*-value < 0.05.

## 3. Results

In this study, 80 children with SHCNs aged 5–16 years were evaluated. The sample comprised 49 males (61.3%) and 31 females (38.7%). The mean age of the participants was 8.44 ± 2.90 years, and their mean weight was 26.1 ± 11.4 kg. Analyses revealed that the groups had statistically similar distributions of sex, age, and weight (Table 1).

The mean duration of the operations was 69.1 ± 26.9 min. There was no significant difference in operation time between the two groups. Additionally, the types of dental procedures, specifically restorative treatments and extractions, did not differ significantly between the groups (Table 2).

In the gastric aspiration performed before extubation at the end of the operation, no blood was observed in the gastric contents of any patient in Group TP. However, blood was observed in the gastric contents of eight patients in Group n-TP, which was significantly higher (*p* = 0.002) (Table 3).

When postoperative sore throat was evaluated, the VAS scores of the patients in Group TP 1 and 2 h postoperatively were significantly higher (*p* < 0.001 and *p* = 0.002, respectively) (Table 4). However, when PONV was evaluated, the PONV values of the patients in Group n-TP 1 and 2 h postoperatively were significantly higher (*p* < 0.001 and *p* = 0.019, respectively) (Table 4).

## 4. Discussion

During dental treatment (periodontal scaling, restorative treatment, and extractions) under general anesthesia, water, blood, saliva, debris, tartar, broken teeth, root pieces, and residual restorative materials are observed in the mouth. Therefore, due to the solid emetic properties of blood in the gastrointestinal system, throat packs are frequently used to minimize the risk of foreign bodies, surgical residues, blood, and other fluid aspiration, despite the cuff of the intubation tube. However, there is still no clear consensus regarding its effectiveness and it may increase postoperative sore throat. The current study aimed to determine the effect of throat packs on PONV and sore throat in children with SHCNs patients. While PONV was statistically significantly higher in Group n-TP patients postoperatively, postoperative sore throat appeared to be higher in Group TP. In this study, the hypothesis “Use of throat packs during dental treatments performed under general anesthesia in children with SHCNs does not affect PONV or postoperative sore throat’’ was rejected.

With the development of modern medicine, the risk of developing dental caries and periodontal disease in children with medical and developmental disabilities has increased, correlating with the increasing survival rates of these children. Dental treatments for children with SHCNs are usually performed under general anesthesia [3]. The advantage of dental treatments under general anesthesia is that all treatments are administered simultaneously without requiring the child’s cooperation. PONV is one of the most common postoperative complications following procedures performed under general anesthesia. In a study conducted on pediatric patients, the incidence of PONV was higher in patients with SHCNs than in healthy patients [3]. Some studies have shown that throat packs do not reduce PONV; however, others argue that they prevent PONV [2,5,13,14,15,16,17,18,19]. A study conducted by Temel et al., which evaluated the effects of throat packs on perioperative gastric volume and PONV in patients undergoing nasal surgery using ultrasonography, concluded that the use of throat packs reduced perioperative gastric volume and was a physical barrier that reduced PONV incidence [20]. In this study, blood was observed in the stomach contents of eight patients in Group n-TP during gastric aspiration before extubation, and only one patient received antiemetics in the postoperative period. Therefore, the fact that the incidence of PONV in the first 2 h after surgery is higher in these patients than in patients who received a throat pack supports that the throat pack is an effective barrier.

However, blood aspiration is not the only cause of PONV. In surgeries performed under general anesthesia, the operation type, duration, anesthetic agents used, and situations that increase gastric insufflation affect PONV incidence. It has been shown that the use of nitrous oxide is not associated with an increased PONV risk and can be used in children [21]. Furthermore, PONV resulting from using inhalation agents is reduced by antiemetic prophylaxis [22], and dexamethasone and methylprednisolone effectively prevent PONV [21]. In this study, methylprednisolone provided antiemetic prophylaxis in all patients.

In pediatric patients, during anesthesia induction, airway pressure and tidal volume cannot be standardized with provider-dependent manual ventilation, which may lead to gastric insufflation resulting in nausea and vomiting [23]. Therefore, limiting peak airway pressure to 15 cmH_2_O is recommended to prevent gastric insufflation during mechanical ventilation in children. In this study, all patients were provided with controlled mechanical ventilation before endotracheal intubation to avoid gastric insufflation, considering the age-appropriate airway pressure and tidal volume. Thus, practitioner-dependent, uncontrolled, high tidal volume and gastric insufflation were prevented.

The cause of sore throat due to treatment under general anesthesia is unknown; however, it is thought to be caused by irritation and inflammation in the trachea due to the endotracheal tube. The endotracheal tube size, cuff inflation pressure, and intubation time may increase the development of irritation and inflammation. Notably, cuff pressures > 30 cmH_2_O can disrupt tracheal mucosal perfusion, leading to necrosis. The recommended cuff pressure is 20 cmH_2_O [24]. In operations performed under general anesthesia, postoperative sore throat associated with endotracheal intubation is observed at approximately 14.4–62% [25]. Throat packs are thought to increase postoperative sore throat, causing trauma and edema in oropharyngeal tissues [5,26]. Postoperative sore throat frequency was 34% in a study evaluating orthognathic surgery cases where cuff pressure was controlled, and throat packing was applied [27]. Similarly, in this study, postoperative sore throat was significantly higher in patients in Group TP. However, the patients did not require postoperative analgesia because the VAS score was ≤5. To the best of the authors’ knowledge, there are no directly comparable studies in the literature, limiting the ability to directly compare the findings.

Postoperative sore throat is correlated with the size of the throat pack; however, there is no study on the specific guidelines regarding optimal sizing. Studies evaluating the relationship between the use of throat packs and sore throat in adults have shown variability in throat pack sizes. In some cases, the size of the throat pack was not reported [14,17,19,20,26,28]. There is existing research on complications associated with using throat packs in children; however, these studies have not successfully evaluated the relationship between throat pack size, sore throat, and PONV [8].

Other than a sore throat, the most common concerns about using throat packs are dysphagia, endotracheal tube displacement, pharyngeal plexus damage, tongue edema, and the risk of being forgotten in place before extubation [29]. Notably, forgetting the throat pack before extubation can lead to very life-threatening consequences. Furthermore, several cases of forgotten throat packs have been reported in the literature [30,31]. The risk of forgetting the throat pack increases due to emergencies that may develop in the operating room, the need for mandatory emergency extubation, or incomplete information transfer during personnel changes. Therefore, various precautions are taken to minimize this risk. However, there are no perfect protocols. The common procedure is to record the throat pack in audio and written forms under the control of the anesthesiologist and the surgeon. In 2009, the UK National Patient Safety Agency published an algorithm that reduced the risk of forgetting a throat pack. The report recommended at least one visual aid (such as labeling the patient or airway, sticking the throat swab to the endotracheal tube, or part of it coming out of the mouth) and at least one documented piece of evidence (such as writing in the operating room) [32]. Similarly, this study used a visual indicator of the throat pack outside the mouth and recorded it verbally and in writing.

Since there are no studies in the literature evaluating the effects of TP use on PONV and sore throat in children with SHCNs, the authors were unable to directly compare their findings. This study was initiated based on prior research; however, a larger sample size may be required to validate the results more robustly. Additionally, an area for improvement in future studies is the standardization of throat pack sizes used across all patients, as the current literature provides insufficient information on this.

## 5. Conclusions

Within the limitations of this study, the use of throat packing in dental treatment of children with SHCNs under general anesthesia reduced the risk of PONV by preventing gastric aspiration. However, it increased the incidence of postoperative sore throat. Therefore, the use of throat packs is recommended for dental treatment under general anesthesia in children with SHCNs due to the increased risk of possible complications related to general anesthesia.

## Figures and Tables

**Table 1:** Patient characteristics.

	Group TP (n = 41) n (%)	Group n-TP (n = 39) n (%)	*p*-Value ^a^
**Sex**			
Male	24 (58.5)	25 (64.1)	0.610
Female	17 (41.5)	14 (35.9)	
	**mean ± SD**	**mean ± SD**	***p*-Value ^b^**
**Age**	8.59 ± 3.2	8.28 ± 2.8	0.808
**Weight**	26.2 ± 10.9	26.1 ± 12.0	0.965

*p*-value < 0.05, a: Ki-kare, b: Mann–Whitney U, SD: standard deviation.

**Table 2:** Distribution of operation time and dental procedures performed by groups.

	Group TP (n = 41) Mean ± SD	Group n-TP (n = 39) Mean ± SD	*p*-Value ^b^
**Operation time**	68.5 ± 26.4	69.6 ± 27.9	0.843
**Dental procedure**			
Restorative treatments	7.40 ± 4.7	6.75 ± 2.5	0.984
Extractions	6.06 ± 3.8	5.17 ± 3.1	0.306

*p*-value < 0.05, b: Mann–Whitney U test, SD: standard deviation.

**Table 3:** Quality of gastric contents.

	Group TP (n = 41) n (%)	Group n-TP (n = 39) n (%)	*p*-Value ^a^
Bloody	-	8 (20.5)	0.002 *
Not bloody	41 (100)	31 (79.5)	

* *p*-value < 0.05, a: Ki-kare.

**Table 4:** Postoperative data.

	Group TP (n = 41) Mean ± SD	Group n-TP (n = 39) Mean ± SD	*p*-Value ^b^
Sore throat, VAS at 1 h	1.32 ± 1.7	0.21 ± 0.9	<0.001 **
Sore throat, VAS at 2 h	0.61 ± 1.1	0.05 ± 0.3	0.002 *
Sore throat, VAS at 4 h	0.17 ± 0.6	0.0 ± 0.0	0.087
PONV at 1 h	0.0 ± 0.0	1.23 ± 1.7	<0.001 **
PONV at 2 h	0.0 ± 0.0	0.26 ± 0.7	0.019 *
PONV at 4 h	0.0 ± 0.0	0.05 ± 0.3	0.305

* p-value < 0.05, ** *p*-value < 0.001, b: Mann–Whitney U test, VAS: Visual Analog Scale, PONV: postoperative nausea and vomiting, SD: standard deviation.

## Data Availability

The datasets used and/or analyzed during the current study are available from the corresponding author on reasonable request. For privacy reasons, however, individual data allowing for the identification of participants cannot be made available.

## Abbreviations

ASA	American Society of Anesthesiologists
BARF	Baxter Retching Faces
ECG	Electrocardiography
Group n-TP	No throat packing
Group TP	Throat packing
NIBP	Noninvasive blood pressure
PONV	Postoperative nausea and vomiting
SHCN	Special healthcare needs
SpO_2_	Oxygen saturation
SPSS	Statistical Package for the Social Sciences
VAS	Visual Analog Scale
VCV	Volume-controlled ventilation
